# *In Vitro* Comparison of Apically Extruded Debris during Root Canal Preparation of Mandibular Premolars with Manual and Rotary Instruments

**DOI:** 10.15171/joddd.2015.026

**Published:** 2015-09-16

**Authors:** Sonal Soi, Suman Yadav, Sumeet Sharma, Mohit Sharma

**Affiliations:** ^1^ Reader, I.T.S Dental College, Hospital and Research Centre, Greater Noida, India; ^2^Professor, Department of Conservative Dentistry and Endodontics, Sri Guru Gobind Tricentenary Dental College, Research Center and Hospital, Gurgaon, India; ^3^Professor and Head of Department, Institute of Dental Studies and Technologies, Modinagar, India

**Keywords:** Apical foramen, root canal, extrusion, manual instrumentation, rotary instruments

## Abstract

***Background and aims.*** During root canal preparation, debris extruded beyond the apical foramen may result in periapical inflammation and postoperative pain. To date no root canal preparation method has been developed that extrudes no periapical debris. The purpose of this study was to identify a system leading to minimal extrusion of debris from the apical foramen. The study was conducted to comparatively evaluate the amount of apical extrusion of debris during root canal preparation using hand ProTaper and GT rotary and RaCe rotary instruments using crown-down technique.

***Materials and methods.*** Ninety freshly extracted human single-rooted mandibular premolars were equally assigned to three groups (n=30). The root canals were instrumented using hand ProTaper, GT rotary and RaCe rotary systems. Debris and irrigant extruded from the apical foramen were collected into vials. The mean weight of the remaining debris was calculated for each group and subjected to statistical analysis.

***Results.*** ANOVA was used to compare the mean dry weights of the debris extruded in the three groups, followedby post hoc Tukey tests for multiple comparisons the between groups. Highly significant differences were found in the amount of debris extruded among all the groups (P<0.001). The ProTaper group exhibited the highest mean debris weight (0.8293±0.05433 mg) and the RaCe system exhibited the lowest mean debris weight (0.1280±0.01606 mg).

***Conclusion.*** All the systems tested resulted in apical extrusion of debris. However, the hand ProTaper files extruded a significantly higher amount of debris than GT and RaCe systems.

## Introduction

Root canal therapy incorporates both cleaning and shaping of the root canal, with the help of instruments and the use of irrigants up to the controlled working length. In spite of maintaining strict working length during root canal preparation, dentin filings, microorganisms, fragments of pulp, necrotic tissue and canal irrigants are extruded beyond the apical foramen.^1,2^ This apical extrusion of debris sometimes results in a foreign body reaction and inflammation of periapical region, leading to intra-appointment or postoperative pain.^2-4^

Apical extrusion of debris has been associated with all types of instruments and instrumentation techniques, even when preparation is maintained short of the apical terminus, with some instrumentation techniques extruding less material than others.^5-8^

Various factors affect the quantity of apically extruded debris, including instrumentation methods, file size and file types.^9^ Instrumentation with irrigants has been shown to produce apical extrusion in contrast to no apical extrusion in instrumentation without irrigants.^7^ The amount of extruded debris has been shown to be directly proportional to the canal length and is strongly influenced by it.^7^ Another study has shown that the amount of apically extruded debris is independent of the canal length.^9^ Manual or mechanical instrumentation with rotary movements reduce the amount of debris by packing dentin chips within the grooves of the file, and thus removing the debris from the root canal.^8^

Conventional hand instrumentation has been shown to extrude more debris when compared to rotary instrumentation. Among the hand instrumentation techniques, step-back instrumentation with circumferential and anticurvature filing has been shown to produce more apical debris when compared to crown-down pressureless and balanced-force techniques.^5,10,11^ Filing (push−pull) motion instrumentation tends to push more debris beyond the apex compared with the use of rotational force. Filing (push−pull) motion creates a plunger action forcing the debris ahead of the file through the patent apical foramen into the periradicular tissues.^5,11-13^ There is less transport of materials with rotary systems compared to hand filing because canal instrumentation with rotary systems remains significantly more centered in the root canal. All these factors indicate that rotary instruments may produce less apically extruded debris than hand instruments.^14-17^ On the contrary, no statistically significant difference has been reported in the apical extrusion of debris by manual and mechanical instrumentation methods.^18^

Root canal preparations with rotary instruments have become popular during the last decade. The hand ProTaper, System GT rotary and RaCe rotary are three contemporary instrumentation systems that are different in their design and use; therefore, differences may also exist between them with regard to apically extruded debris. The purpose of this study was to compare and evaluate the amount of apically extruded debris using these three systems.

## Materials and Methods

A total of 90 freshly extracted human single-rooted mandibular premolar teeth with complete root formation were selected.

Teeth with one root canal and one apical foramen, with canal curvature between 0 and 10 degrees and an apical diameter corresponding to #10 K-file, were selected. Periapical radiographs were taken to confirm that all the samples had a patent single root canal with single apical foramen. The curvature of the root was determined using Schneider’s technique and only teeth with curvatures from 0 to 10 degrees were included to eliminate the complications likely to occur in a severely curved root canal.^12,19^ Teeth with calcification and open apices were excluded.

The teeth were cleaned of debris and soft tissue remnants and stored in physiological saline solution until required. The cusps of the teeth were flattened using a high-speed handpiece bur. Endodontic access cavities were prepared (Endo Access Bur, Dentsply Maillefer, Ballaigues, Switzerland) in a high-speed handpiece and pulpal remnants were extirpated using a broach.

The working length was determined by placing a #15 K-file until it was just visible at the apical foramen. From this, 1 mm was subtracted to determine an accurate working length. The working length for each sample was recorded.

### Preparation of Test Apparatus

**Figure 1.**
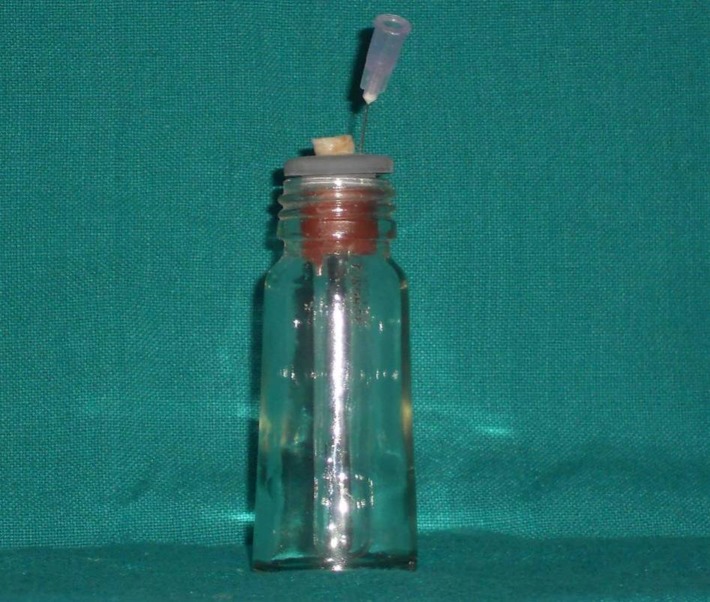
Test apparatus for debris collection.

The debris and irrigant collection apparatus was similar to that described by Myers and Montgomery (Figure 1).^6^ Ninety glass tubes were preweighed to 10-^2^mg precision using an electronic microbalance (Dhona 100 DS, Dhona Instruments (P) LTD., India - single pan analytical balance with resolution of ±0.01 mg and a weighing capacity of 100 mg). They were used as the containers for collecting any extruded debris or irrigants and were placed in a glass flask with the rubber stopper fitted securely onto the mouth of the flask. Each tooth was secured by its root being forced through a precut hole in the rubber stopper. The apex of the root was suspended below the upper rim of the collection vial. A 25-gauge needle was placed alongside the stopper during insertion to equalize the air pressure inside and outside the flask. The tubes were numbered. A rubber dam was placed to prevent any bias by the practitioner. This simulated the clinical working environment where the operator is dependent on working length determined by radiographs or electronic apex locators without visualizing the root apex.

### Quantification of Debris

The debris adhering to the outer surface of the root apex was collected by washing the apex with an additional 1 mL of distilled water into the tube. All the glass tubes were stored in an incubator at 68°C for two days to evaporate the irrigant before weighing the dry debris.^20^ The tubes containing the dry debris were weighed. Three consecutive measurements were taken and the average was recorded to 10^-2^mg precision using an electronic microbalance (Dhona 100 DS). The weight of extruded debris was calculated by subtracting the weight of the preweighed empty tube from the weight of the tubes plus the dried debris:^6,20^

Weight of extruded debris = (weight of the tube + dry debris) – weight of the tube

The samples were equally divided into three groups of 30 samples each for instrumentation with different techniques. A volume of 2 mL of distilled water was used after each instrument for irrigation. Patency of the canal was maintained with a #10 K-file after each instrument.^21^ The crown-down technique was used during the instrumentation process and shaping was carried out to the working length according to the protocol described by the manufacturer.

Group A (n=30): Hand ProTaper files (Dentsply Maillefer, Ballaigues, Switzerland). A set of six instruments were used: three shaping files (Sx, S1, and S2) for a crown-down procedure and three finishing files (F1, F2, and F3) for apical shaping.

Group B (n=30): System GT rotary files (Dentsply Maillefer, Ballaigues, Switzerland) were used with a gear-reduction, torque-controlled, contra-angle handpiece at 300 rpm. Shaping of the root canals was carried out using files in the following crown-down sequence: 30/.10, 30/.08, 30/.06, 30/.04, and 30/.06.

Group C (n=30): RaCe rotary files (Reamer with Alternating Cutting Edges, FKG Dentaire, La-Chaux-de-Fonds, Switzerland) were used with a gear-reduction, torque-controlled, contra-angle handpiece at 500 rpm. The root canals were shaped using the following crown-down sequence: 25/.06, 20/.06, 30/.04, 25/.04, 20/.02, 25/.02 and 30/.02.

### Statistical Analysis

SPSS 17 for Windows was used for analyzing the results. One-way ANOVA was used to compare the mean dry weights of the debris extruded in the three groups. A P-value <0.05 was considered to be significant. Multiple comparisons by post hoc Tukey HSD (Honest Significant Difference) tests were performed for comparisons between the three sub-groups (hand ProTaper/System GT rotary; hand ProTaper/RaCe rotary; System GT rotary/RaCe rotary).

## Results

**Figure 2.**
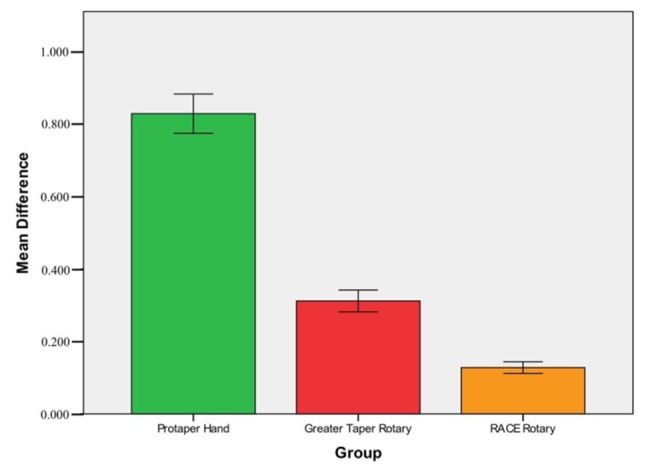
Bar Diagram showing comparison of apically extruded debris in hand ProTaper, System GT rotary and RaCe rotary instruments.

Hand ProTaper system (0.8293±0.05433 mg) had the highest mean extrusion of debris, followed by System GT rotary (0.3120±0.03022 mg) and RaCe rotary systems (0.1280±0.01606 mg) in descending order (Table 1). One-way ANOVA with respect to the amount of apically extruded debris by three different root canal instrumentation techniques demonstrated significant differences between the three groups in relation to the weight of debris extruded (P<0.001) (Table 2). Post Hoc Tukey HSD tests between the three sub-groups (hand ProTaper/System GT rotary; hand ProTaper/RaCe rotary; System GT rotary/RaCe rotary) showed differences between the subgroups (Table 3). Instrumentation with RaCe system produced the least amount of extruded debris, which was significantly lower compared to ProTaper and GT systems (Figure 2).

**Table 1:** Mean extruded debris by the three different root canal instrumentation techniques

**Group**	**N**	**Mean**	**SD**	**95% Confidence interval for mean**	
				Lower bound	Upper bound
Hand ProTaper	30	0.8293	0.05433	0.8090	0.8496
System GT Rotary	30	0.3120	0.03022	0.3007	0.3233
RaCe Rotary	30	0.1280	0.01606	0.1220	0.1340

**Table 2:** One-way ANOVA with respect to the amount of apically extruded debris by three different root canal instrumentation techniques

		**Mean Square**	**F**	**Significance**
	Between Groups	0	2886.829	<0.001
Weight of extruded debris	Within Groups	0		
	Total			

**Table 3:** Multiple comparisons using post hoc Tukey tests between the sub-groups (hand ProTaper/System GT ro-tary; hand ProTaper/RaCe rotary; System GT rotary/RaCe rotary)

**Comparison**	**Mean Difference**	P value^**^
Hand ProTaper vs. System GT rotary	0.5173	<0.001
Hand ProTaper vs. RaCe rotary	0.7013	<0.001
System GT rotary vs. RaCe rotary	0.1840	<0.001
	^** P<0.001; highly significant^

## Discussion

Several factors affect the amount of intracanal materials extruded, including the tooth, instrumentation technique, instrument type, size and preparation end point, and irrigation solution.^5,8,12-14,22,23^ The type of tooth plays a very important role. In previous studies, single-rooted mandibular premolars,^8,24^ single-rooted maxillary lateral incisors and mandibular premolars,^6^ maxillary and mandibular central and lateral incisors with single canals,^14^ maxillary anterior teeth,^23^ and single-rooted anterior teeth and premolars have been used.^25^

In the present study, only single-rooted mandibular premolars were used because application of one kind of tooth can help increase the similarity between specimens. The teeth were carefully selected according to the tooth type, canal size, working length and canal curvature. The teeth were radiographed from clinical and proximal aspects to ensure that they had single canals and single orifices.

Apical patency was maintained with a #10 K-file in all the cases to achieve standardization of apical diameters. This was done to provide a standardized diameter with respect to extrusion in samples.^21^ Tinaz et al^22^ showed that as the diameter of apical patency increased, the debris extrusion also increased, while Lambrianidis et al^1^ paradoxically reported that greater amount of extrusion occurred when the apical constriction remained intact. Two other studies, however, found no correlation between the amount of debris extruded and apical diameter.^9,10^ Greater amount of debris extrusion occurred when instrumentation was carried out beyond the foramen.^26^

Irrigation is a necessary and important phase of root canal cleansing procedures. Fairbourn et al^24^ used tap water; however, tap water left some salt residues on drying, which may have added to the weight of extruded debris; thus it was not used in our study. Sodium hypochlorite as the most popular irrigant was used in a pilot study before initiating the present investigation. However, on drying, it resulted in salt crystal formation and added to the weight of debris extruded. Thus, it affected the accuracy of measurements of the apically extruded debris.^5,6,9,27^ Hence, in the present study distilled water was selected as an irrigant; it was injected passively over 15 seconds using a monojet irrigating syringe to minimize forcing the debris out of the apical foramen.^10,18,21,28^

Many studies,^15,20,29^ including ours, have measured only the amount of debris extruded. However, in some other studies the extrusion of debris and irrigants has been considered because both of them are responsible for periapical inflammation, postoperative pain and possible delayed healing.^13,14,30,31^

In our study, hand ProTaper system exhibited the highest mean extrusion of debris (Tables1 and 3) (Figure 2). The results were consistent with previous studies that have demonstrated that ProTaper system extrudes larger amounts of apical debris compared to other instrumentation systems.^12,15,20,32^ The ProTaper, being a faster system with fewer instruments, removes substantial amounts of dentin in a short period because of its greater cutting capacity, long pitch design, and taper.^20,33-35^This may result in greater apical extrusion of debris.^20,29,36^ Furthermore, hand ProTaper has been shown to extrude greater amount of debris when compared to ProTaper rotary system. This might be attributed to the fact that hand ProTaper files prepare the apical area for an extended period of time and the rotational movement of the file is a variable factor that is controlled by the operator.^20,37,38^ However, hand ProTaper files may display lower apical extrusion compared to the ProTaper rotary with the use of modified balanced-force technique. The controlled pressure of the instrument inside the root canal with this technique allows for efficient removal of debris adhering to the files.^8,20^ To the best of our knowledge very few studies have reported the use of hand ProTaper instrument design.^8,20,37^

System GT rotary files produced the second highest amount of apical debris extrusion (Tables1 and 3) (Figure 2). They possess a slight negative rake angle and radial lands that make them cut less aggressively than those having an active cutting blade. Their unique 'U' file design encourages coronal rather than apical displacement of debris.^35,39,40^ These features may account for lower apical debris extrusion when compared to the hand ProTaper system. To the best of our knowledge, no study has evaluated the amount of apically extruded debris with System GT rotary files. However, one study has evaluated the apical extrusion of intracanal bacteria using System GT.^41^

The amount of debris extruded with RaCe was the lowest among the three groups (Tables1 and 3) (Figure 2). Our results were consistent with other studies where instrumentation with RaCe system produced less debris.^13,15,32^ The RaCe system possesses features that may account for reduced debris extrusion, presumably due to its non-convex triangular cross-sectional design and smaller core diameter, which allows more space to carry debris towards the root canal orifices. Furthermore other design features of RaCe, such as its short twisted cutting edges alternating with straight edges, may give rise to favorable debris-transporting spaces.^15,42^

An instrumentation method that extrudes no periapical debris has not yet been developed. It has been demonstrated that debris extruded from the apical foramen may be related to mid-treatment or post-treatment flare-up phenomena.^14^ Furthermore, the periapical extrusion of microbes into the periradicular tissues during endodontic treatment has the potential to bring about serious systemic diseases, such as endocarditis, brain abscesses, septicaemia and endodontic cellulitis, particularly in medically compromised patients.^3,36,37,43^ Thus, an instrumentation technique that minimizes apical extrusion of debris would be advantageous to both the clinician and the patient.

The results of this study indicated that practitioners should be aware of the extent of debris extrusion with each specific instrument, which can probably be made the basis for selection of a particular instrument. Restriction of the hand ProTaper to vital and less infected teeth is one possible measure that can be taken into account to prevent acute ﬂare-ups. Similarly, the RaCe system can be used for chronic, heavily infected root canals and in teeth with resorbed apices due to the lower extrusion of apical debris.

It must be emphasized that the results of this study should not be directly applied to clinical situations. In our in vitro study, the tooth apex was suspended in air, whereas in vivo the apex would be surrounded by granulomatous or periradicular tissues, which might serve as a natural barrier, restricting extrusion of debris to some extent.^7,44-46^ The results might also differ because of positive and negative pressures at the apex.^45^ Furthermore, mid-treatment ﬂare-up may not only depend on the amount of debris extruded, but also it may depend on the amount of extruded irrigant, intracanal medication, bacterial virulence and the host response.^12^ It might be possible that the smaller amount of debris extruded by one type of instrumentation system may contain organisms of higher virulence and antigencity, when compared to instruments extruding larger amount of debris. Thus in this condition, there may be flare-up even with comparatively smaller amount of debris with the selected instrumentation techniques. As this study was limited to teeth with mature root morphology, the results should not be generalized to teeth with immature root development and open apices.

## Conclusion

Within the limitations of this study, it can be concluded that all the three instruments tested (ProTaper, System GT and RaCe) resulted in some apical extrusion of debris. The hand ProTaper extruded a significantly higher amount of debris among the three instruments.

## Acknowledgments

Mr. Gurinder Singh for statistical analysis.
